# An efficient system for intracellular delivery of beta-lactam antibiotics to overcome bacterial resistance

**DOI:** 10.1038/srep13500

**Published:** 2015-08-27

**Authors:** Nadia Abed, Fatouma Saïd-Hassane, Fatima Zouhiri, Julie Mougin, Valérie Nicolas, Didier Desmaële, Ruxandra Gref, Patrick Couvreur

**Affiliations:** 1 Faculty of Pharmacy, Institut Galien UMR CNRS 8612, University of Paris-Sud XI, 5 Rue Jean-Baptiste Clément, 92296 Châtenay-Malabry Cedex, France; 2 Faculty of Pharmacy, INSERM IFR 141 University of Paris-Sud XI, 5 Rue Jean-Baptiste Clément, 92296 Châtenay-Malabry Cedex, France

## Abstract

The “Golden era” of antibiotics is definitely an old story and this is especially true for intracellular bacterial infections. The poor intracellular bioavailability of antibiotics reduces the efficency of many treatments and thereby promotes resistances. Therefore, the development of nanodevices coupled with antibiotics that are capable of targeting and releasing the drug into the infected-cells appears to be a promising solution to circumvent these complications. Here, we took advantage of two natural terpenes (farnesyl and geranyl) to design nanodevices for an efficient intracellular delivery of penicillin G. The covalent linkage between the terpene moieties and the antibiotic leads to formation of prodrugs that self-assemble to form nanoparticles with a high drug payload between 55–63%. Futhermore, the addition of an environmentally-sensitive bond between the antibiotic and the terpene led to an efficient antibacterial activity against the intracellular pathogen *Staphylococcus aureus* with reduced intracellular replication of about 99.9% compared to untreated infected cells. Using HPLC analysis, we demonstrated and quantified the intracellular release of PenG when this sensitive-bond (SB) was present on the prodrug, showing the success of this technology to deliver antibiotics directly into cells.

Intracellular infections are important public health concern; they are recurrent, persistent and difficult to treat. They are generally produced by intracellular bacteria, such as *Staphylococcus aureus*, *Mycobacterium tuberculosis* or *Salmonella* Typhimurium and commonly associated with human diseases. The intracellular localisation of those bacteria provides them protection against the host immune system and action of antibiotics, leading to recurent infections^1^,^2^. In fact, some antibiotic families (i.e. β-lactams or aminoglycosides)^3^ fail to accumulate efficiently into eukaryotic cells while others (i.e. macrolides or fluoroquinolones), exhibit poor retention inside the cells^4^,^5^. Therefore, the antibiotic concentration in the intracellular compartment is often sub-therapeutic, resulting in a drastic reduction in their effectiveness against intracellular pathogens, but also in promotion of antibiotic resistance^6^.

Thus, the treatment of intracellular bacterial infections still remains a major pharmaceutical challenge, despite a large panel of antibiotics already on the market. With the increase of multidrug resistant bacteria, it becomes urgent to improve the antibiotics transport through the cell membrane. To overcome this issue, the development of nanocarriers able to deliver antibiotics directly into infected cells may represent a promising solution^7^. In addition, these systems may also protect the antibiotic from degradation and eventually allow its controlled-release directly nearby the pathogen, therefore achieving therapeutic levels at the foci of infection and avoiding side effects.

Over the past decades, a plethora of drug delivery systems has been described in many medical areas, including intracellular infections^8^,^9^,^10^. An interesting feature with intracellular infection is that pathogenic bacteria establish their niche mainly into phagocytic cells like macrophages. It is well known that, when injected intravenously, liposomes/nanoparticles (NPs) are rapidly recognized and taken out from the circulation by phagocytic cells of the reticuloendothelial system (RES), thereby targeting “passively” the reservoir/sanctuary of most intracellular pathogens^8^. Among these systems already described, Lecaroz *et al.* used poly(lactide-co-glycolide) acid (PLGA) to design gentamicin loaded NPs, in order to treat *in vitro* and *in vivo* infections caused by the intracellular pathogen *Brucella* spp^11^. Similarly, previous studies in our laboratory have also shown that ampicillin loaded onto polycyanoacrylate (PACA) NPs efficiently reduced *Salmonella* Typhimurium infection *in vitro* and *in vivo,* but without eradicating the infection^12^. However, the major drawback of those nanocarriers is their poor drug payload (weight of the drug/weight of the loaded-NPs_(drug+carrier)_)^13^,^14^,^15^ which is especially detrimental for antibiotherapy when high dose regiments are needed in case of severe, chronic and/or resistant infections^16^,^17^. This implies administration of a large amount of the carrier material in order to achieve sufficient drug concentration at the site of infection, raising toxicological concerns. Moreover, other issues such as instability of the nanocarrier and “burst release”^18^ of the antibiotic are also hurdles which explain in part, why that no antibiotic nanomedicine has reached the market until now. Therefore, it is needed to design “nanoantibiotics” with: (i) high drug payload, (ii) absence of “burst release” and (iii) specific antibiotic delivery in the infected intracellular sanctuaries.

The linkage of penicillin G to squalene, a natural and biocompatible lipid, has recently allowed us to reach some of the above mentioned requirements since the resulting bioconjugate self-assembled spontaneously as stable nanoparticles and displayed a high drug payload (ca. 45%)^19^. It was shown that these squalene-based NPs displayed a better intracellular antibacterial activity than the free antibiotic. However, the intracellular antimicrobial activity was associated with cell toxicity, probably due to the intracellular hydrolysis of the squalene-penicillin G bioconjugates, leading to the formation of squalenic acid, a strongly surface-active compound^19^,^20^. It is worth to point out that squalene belongs to the terpenes family, a group of organic molecules composed of over 30,000 isoprenoid natural compounds (consisting of isoprene units arranged in a specific pattern) which are extraordinary diverse in structure and biological function^21^,^22^. Thus, our approach was to select among this family, polyisoprene chains with shorter lengths (i.e. geranyl and farnesyl chains, with respectively two and three isoprenic units) in order to decrease NPs cytotoxicity, since shorter are the corresponding acids, lower should be their surface active property. Moreover, in case where the molecular weight (Mw) of the terpenic moiety (carrier material) is reduced compared with the drug’s Mw, the NPs drug payload should further be improved. Finally, we have used a environmentally-sensitive chemical bond (SB) between terpene and penicillin G to design an nanoantibiotics, allowing release of the antibiotic in the intracellular compartments of infected-cells. Both, geraniol and farnesol are, just as squalene, natural precursors in the cholesterol’s biosynthesis and other steroids, making them good biodegradable carriers.

This paper describes the synthesis of the geranyl and farnesyl penicillin G (PenG) bioconjugates with or without a sensitive-bond (SB) as well as their formulation process as nanoparticles (NPs). The cytotoxicity profiles of the different NPs, their cell internalisation, their antimicrobial activity on *Staphylococcus aureus*-infected macrophages and finally, the intracellular penicillin G release were also investigated in order to identify the best candidate among the four nanoparticles.

## Results

### Synthesis of bioconjugates

**Figure 1:**
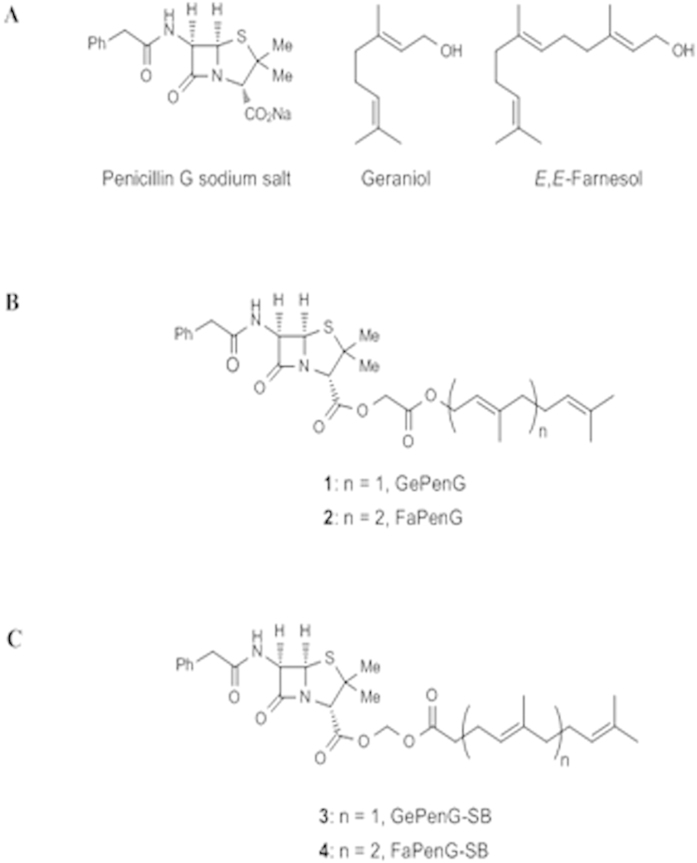
(**A**) Chemical structure of the different moieties: Penicillin G sodium salt (PenG), Geraniol and E,E-Farnesol. (**B**) Chemical structures of the GePenG (**1**), FaPenG (**2**) biocongugates. (**C**) Chemical structures of the bioconjugates harbouring an environmentally-sensitive bond between the terpene fraction and the penicillin G: GePenG-SB (**3**)and FaPenG-SB (**4**).

All the bioconjugates were synthesised and characterised as fully described in supplementary information. Briefly, bioconjugates **1** and **2** displaying two (geranyl) and three (farnesyl) isoprene units were synthesised through direct alkylation of the carboxylate group of benzyl penicillin G sodium salt (PenG; Fig. 1A) with geranyl and farnesyl bromoacetates, respectively (leading to GePenG and FaPenG bioconjugates) (Fig. 1B). In these conjugates, a simple ester bond connects PenG with either Ge or Fa moieties. Since the cleavage of the carrier is a key issue for the biological activity, an environementally-sensitive acylal linker was also intercalated between the polyisoprenyl moiety and the PenG, leading to GePenG-SB and FaPenG-SB. These derivatives (GePenG-SB (3), FaPenG-SB (4)) were prepared by alkylation of the carboxylate group of Penicillin G with geranyl- and farnesyl acetic acid chloromethyl esters (Fig. 1C).

### Formulation and physicochemical characterisation of nanoparticles

First, the nanoparticles were formulated with a simple method of nanoprecipitation followed by a step of solvent evaporation^19^,^23^. Farnesyl- and geranyl-penicillin G bioconjugates (FaPenG and GePenG) as well as their corresponding sensitive-bond derivatives (FaPenG-SB and GePenG-SB) spontaneously self-assembled as narrowly-dispersed nanoparticles at a concentration of 4 mg.mL^−1^ in water. Then, we used dynamic light scattering (DLS) and electrophoretic light scattering which represent the two main techniques commonly employed to determine respectively, the size (and the size distribution) and the surface charge of particles in a colloidal suspension. As summarized in Table 1, FaPenG nanoparticles displayed an average diameter of 259 nm and a negative zeta potential (ζ-potential) of −47 mV while FaPenG-SB NPs had a smaller size (195 nm) and a ζ-potential of −61 mV. The smaller analog GePenG-SB displayed similar size and zeta potential (diameter = 250 nm and ζ-potential = −63 mV), whereas GePenG NPs, exhibited a less negative ζ-potential (−20 mV) and formed significantly larger nanostructures (464 nm). In all cases, NPs exhibited very low polydispersity indexes, ranging from 0.04 to 0.09, meaning that the nanoparticles were rather uniform in size (Supplementary Fig. S2). Furthermore, by decreasing the terpene chain length, the NP’s drug content was consequently augmented reaching 63 wt% (Table 1), which is well beyond the drug payload of classical drug delivery systems, such as liposomes or polymeric nanoparticles. This parameter is calculated as follows: Drug content = [(Mw_PenG_ × 100)/Mw_NPs_], where Mw_PenG_ is the Molecular weight of Penicillin G and Mw_NPs_ is the Mw of the considered bioconjugate. Interestingly, an increase in the NPs diameter (FaPenG < GePenG) correlated well with a reduction of the isoprenic chain length (Fa > Ge). The NPs stability has been also tested during 24 hours incubation (at 37 °C) in serum-enriched cell culture media; apart for GePenG NPs which displayed a rapid increase in size, all other NPs formulations were rather stable, even if a small decrease in size was noticed for the GePenG-SB NPs. In fact, GePenG NPs behave differently than the other formulations, since either in water or in serum-enriched cell culture media, the size of those NPs increased up to ≥800 nm, after only a few hours (Supplementary Fig. S3 and Fig. S4). It is known that a higher surface charge of NPs improves stability in solution, because of the strong repellent forces among particles. GePenG NPs displayed a lower absolute ζ-potential than the other NPs, which may partly explain their relative instability. By using standard optical microscopy analysis, we observed that after 24 h in water, the GePenG NPs suspension was composed of individualized spherical NPs with a diameter ≥1 μm (Supplementary Fig. S5A), suggesting that the NPs coalesce over time in solution.

**Figure 2:**
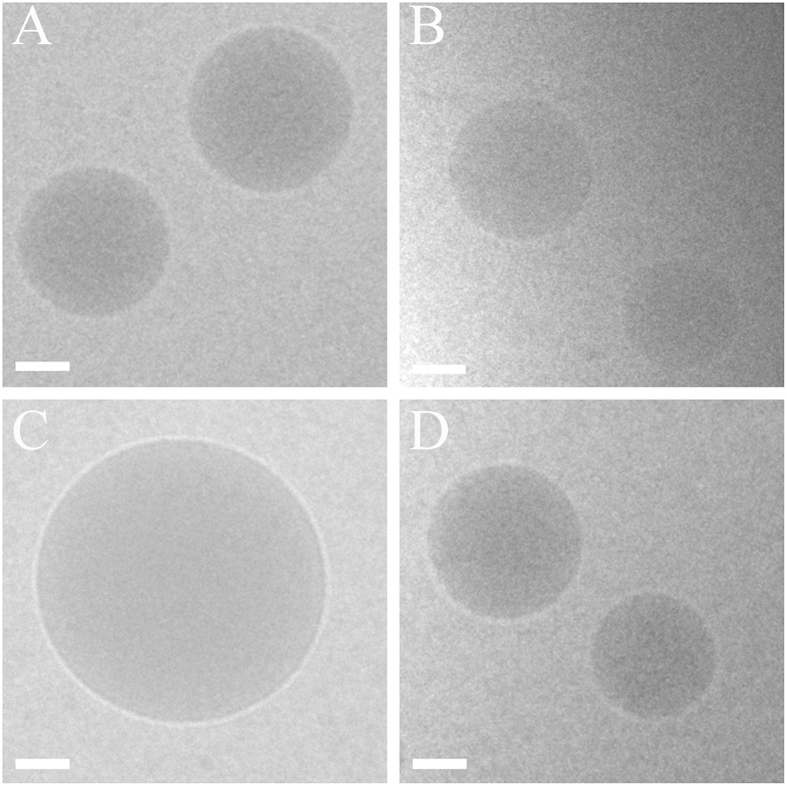
Cryogenic transmission electron microscopy images of FaPenG (**A**), FaPenG-SB (**B**), GePenG (**C**) and GePenG-SB (**D**) nanoparticles. Scale bars = 100 nm.

CryoTEM morphological analysis of FaPenG, FaPenG-SB and GePenG-SB NPs showed a dense spherical shape devoid of any observable supramolecular structures; NPs sizes were in good agreement with dynamic light scattering (DLS) measurements (Fig. 2). Concerning GePenG NPs, they exhibited a spherical shape, too (Fig. 2c), but with a tendency to coalesce together as clearly observed on supplementary Figure S4B, confirming their instability. Since additional assays didn’t succeed to improve neither the size nor the colloidal stability of GePenG NPs, they were not further used in the biological studies.

### Growth inhibition assay on *Staphylococcus aureus*

To check the antibiotic activity of Penicillin G after conjugation to short terpenic chains, *Staphylococcus aureus* ATCC55585, a penicillin G-sensitive strain, was chosen. This bacterium is representative of facultative intracellular bacteria with phagolysosomal localisation^1^,^24^. First, we carried out a basic experiment to determine the MIC (minimal inhibitory concentration, i.e. the lowest concentration required for complete inhibition of bacterial growth) of geranyl and farnesyl NPs against *Staphylococcus aureus*. Practically, a wide range of FaPenG, FaPenG-SB and GePenG-SB NPs concentrations were directly incubated with a suspension of *S. aureus* overnight at 37 °C. The results presented in Table 2, clearly show that NPs harbouring a sensitive linker (i.e. FaPenG-SB and GePenG-SB) displayed an antimicrobial activity similar to free PenG (0.03 μg.mL^−1^), with MIC of 0.15 and 0.1 μg.mL^−1^, respectively. Since no antimicrobial activity was observed with FaPenG NPs (even at concentration of 225 μg.mL^−1^), it was suggested that the sensitive linker was required to release the antibiotic from NPs (Supplementary Fig. S6). This may be explained by the fact that, during their growth, bacteria secrete a lot of molecules and enzymes such as lipases or proteases^25^,^26^, thus creating an environment that could trigger PenG release from nanoantibiotics only when they were equiped with the sensitive-bond. It is worth noting that, when the hydrolysis of FaPenG-SB and GePenG-SB occurs, both penicillin G and farnesyl/geranylacetic acids are produced. Therefore, these two compounds (FaCH_2_CO_2_H and GeCH_2_CO_2_H) were further formulated as NPs and checked for absence of antimicrobial activity using MIC assay (Table 2).

These data strongly suggested that the antimicrobial activity of FaPenG-SB and GePenG-SB NPs directly resulted from the antibiotic release without any significant influence of the terpenic chain length on the antimicrobial activity.

### *In vitro* cytotoxicity

Phagocytic cells being known as main sanctuaries for intracellular pathogens, RAW 264.7 mouse macrophages were used for NPs cytotoxicity investigations (using MTT assay)^7^,^27^. Moreover, this cell line was further used as cell host for *Staphylococcus aureus* infection studies. Practically, cells were incubated at 37 °C with increasing concentrations of FaPenG, FaPenG-SB or GePenG-SB NPs in serum-enriched culture cell media and after 24 h, cell viability was estimated. As shown in Table 3, FaPenG-SB NPs displayed higher cytotoxicity (IC_50_ ≈ 22 μg.mL^−1^) than FaPenG (IC_50_ ≈ 112 μg.mL^−1^) and GePenG-SB NPs (IC_50_ ≈ 72.5 μg.mL^−1^). As a matter of comparison, we also tested the cytotoxicity of SqPenG-SB NPs and confirmed a similar cytotoxicity profile (IC_50_ ≈ 18 μg.mL^−1^) to the one observed with FaPenG-SB NPs (Table 3). The higher cytotoxicity observed with FaPenG-SB and SqPenG-SB NPs, comparatively to GePenG-SB NPs, could be attributed to the release of farnesylacetic or squalenic acid which are known to have higher surfactant properties than geranylacetic acid on cellular membranes, likely in destabilizing them. The fact that the FaPenG NPs without a sensitive-bond were less cytotoxic supports this hypothesis. It is worth mentioning that the cytotoxic effect of squalenic acid has already been observed against murine melanoma and human colon carcinoma cells at similar range of concentrations^20^. Finally, we checked the PenG cytotoxicity on RAW cells and as expected, no cytotoxic effect was observed (Table 3).

### Cell internalisation

**Figure 3:**
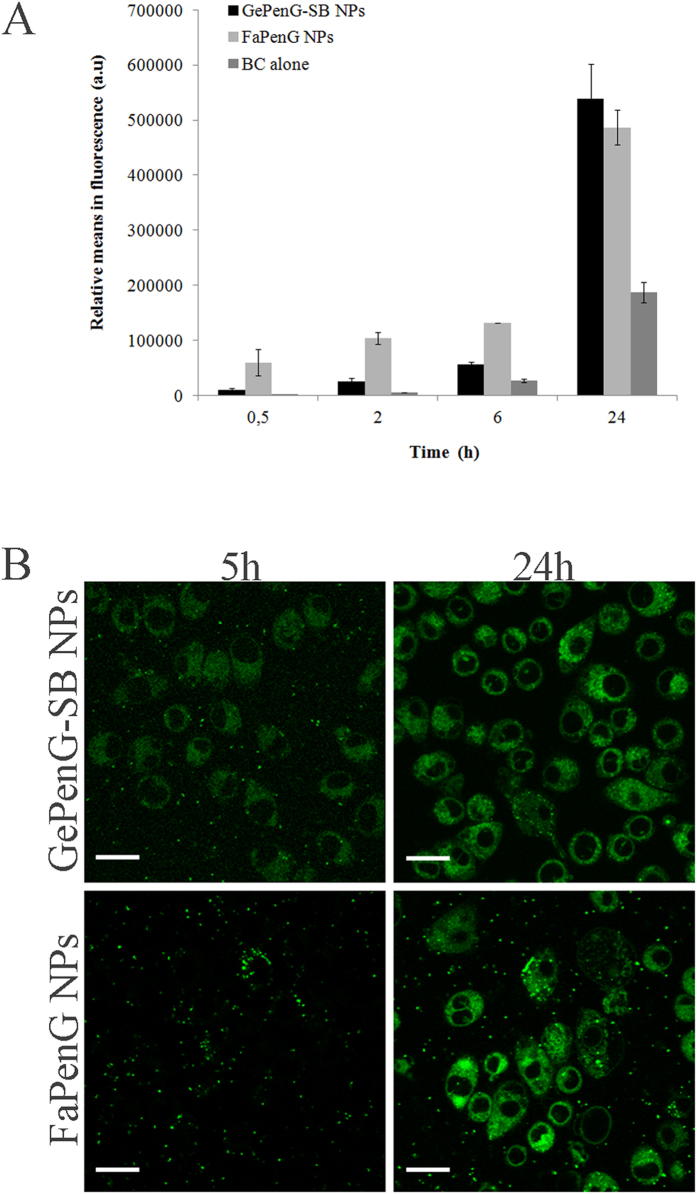
Cell uptake of NPs by RAW 264.7 cells. (**A**) Kinetics of uptake of fluorescently labelled NPs (FaPenG::BC and GePenG-SB::BC) and free probe (BC alone) by flow cytometry. (**B**) Confocal microscopy of RAW 264.7 cells treated (5 h and 24 h) with fluorescent NPs (FaPenG::BC and GePenG-SB::BC). NPs were fluorescently labelled in green with 0.5% BODIPY® FL C12 cholesteryl ester. Scale bars = 20 μm.

The uptake of FaPenG and GePenG-SB NPs by RAW 264.7 macrophages has been investigated using fluorescently labelled NPs. Green fluorescent FaPenG, FaPenG-SB and GePenG-SB NPs (respectively named: FaPenG::BC, FaPenG-SB::SB and GePenG-SB::BC) were obtained by co-nanoprecipitation of the bioconjugates (i.e. FaPenG, FaPenG-SB or GePenG-SB) with 0.5% BODIPY® FL C12 cholesteryl ester (green fluorescent probe). The labelling procedure didn’t significantly change NPs diameter and zeta potential (Table 1). We initially investigated the kinetics of NPs internalisation by flow cytometry analysis of FaPenG and GePenG-SB NPs. As shown in Fig. 3A, both NPs were efficiently taken-up by RAW 264.7 macrophages but FaPenG NPs entered the cells more rapidly than GePenG-SB NPs. However, after 24 hours incubation, cells displayed similar intracellular fluorescence when treated with either FaPenG or GePenG-SB NPs (Fig. 3A). Moreover, after 24 h, the free probe did not accumulate into cells as much as the NPs. The intracellular distribution of fluorescently labelled NPs was further investigated by confocal microscopy to ensure that NPs were inside of the cells rather than absorbed onto the cell membrane (Fig. 3B). This has been shown by using CD11b-APC antibody as a red marker of the cell membrane, which didn’t overlap with the green fluorescence of NPs (Supplementary Fig. S7). Finally, by using time-lapse confocal microscopy on living cells, we observed that both GePenG-SB and FaPenG NPs entered into the macrophages as intact nanoparticles; already after 2 h of incubation, fluorescent NPs were clearly visible inside the cells (Movie M1 and M2). However, after 5 h of incubation, the distribution of the intracellular fluorescence dramatically varied depending on NPs considered: FaPenG NPs displayed clear intense spots inside of the macrophages which suggested that these NPs stayed intact at this time point, while GePenG-SB NPs exhibited more diffuse fluorescence, signifying that they unravelled more rapidly than FaPenG NPs (Movie M3 and M4). After 24 h, both NPs seemed to unravel homogenously into cells and, as already observed by FACS analysis, the intracellular fluorescence was rather the same (Fig. 3B). Since FaPenG-SB NPs have proven to be cytotoxic in RAW cells (MTT assay), we could not use them for further internalisation studies. Using videomicroscopy analysis, the behavior of RAW cells treated with FaPenG-SB::BC NPs over 24 h has been investigated. As shown in movie M5, cells didn’t survive to the treatment with fluorescent FaPenG-SB NPs. Since the principal aim of this study was to design and select an effective drug delivery system with high drug payload but without cytotoxicity, FaPenG-SB NPs could not be longer considered as good candidates for further antimicrobial efficacy studies. In addition, results would be skewed by cellular cytotoxicity.

### Intracellular killing

**Figure 4:**
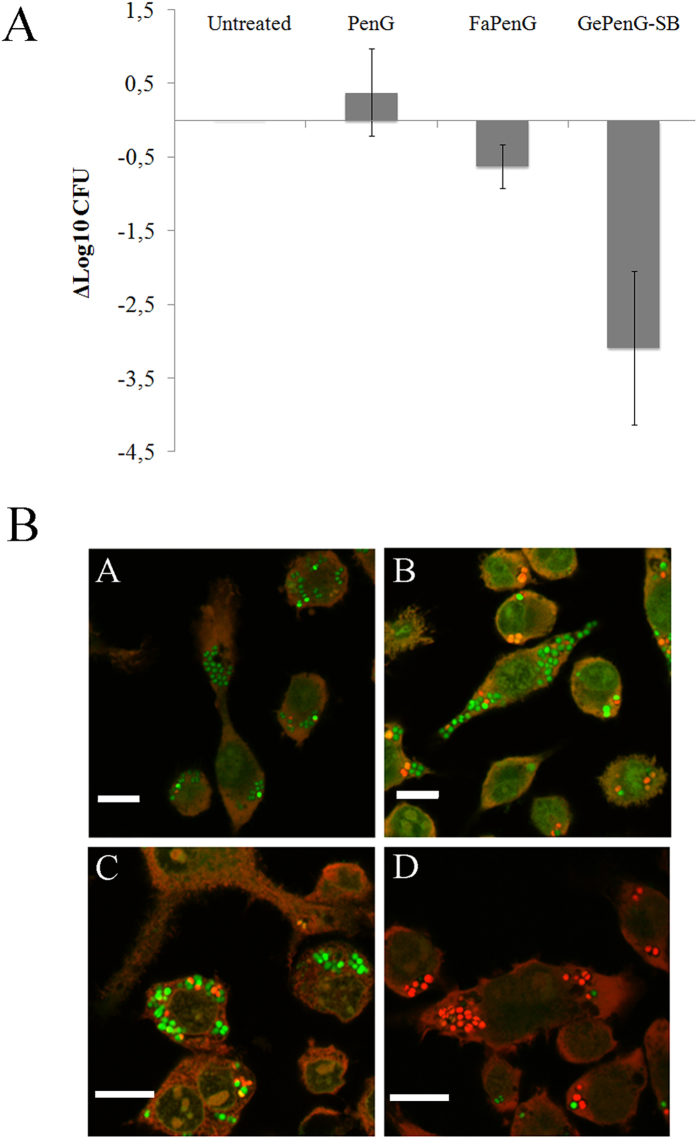
Intracellular survival of *Staphylococcus aureus* in RAW 264.7 cells after NPs treatment. (**A**) Infected RAW 264.7 macrophages were incubated (20 h) or not with PenG, FaPenG NPs or GePenG-SB NPs at 20 μg.mL^−1^, equiv. PenG. Control was untreated infected-macrophages. (**B**) Confocal microscopy of viable/dead intracellular bacteria (*S. aureus*) in RAW264.7 cells after 6 h treatment with PenG (**B**), FaPenG NPs (**C**) or GePenG-SB NPs (**D**) at 20 μg.mL^−1^, equiv. PenG. Control was untreated infected-macrophages (**A**). After gentamicin treatment, cells were stained with LIVE/DEAD BacLight® kit. Living intracellular bacteria are labelled in green. Dead intracellular bacteria are labelled in red. Scale bars = 20 μm.

The antimicrobial activity of GePenG-SB and FaPenG NPs was tested *in vitro* against *Staphylococcus aureus*-infected macrophages and compared to free PenG. Practically, RAW 264.7 cells were first infected by *S. aureus* at multiplicity of infection (MOI) of 10, during 90 min. After cell washing and killing the remaining extracellular bacteria, cells were treated with 20 μg.mL^−1^ (equivalent PenG) of FaPenG NPs, GePenG-SB NPs or bare PenG. Figure 4A illustrates the antimicrobial activity obtained after 20 h of treatment. Neither FaPenG NPs nor free PenG displayed significant antimicrobial activity, confirming the resistance of intracellular *S. aureus* to Penicillin treatment. On the contrary, the treatment with GePenG-SB NPs resulted in a spectacular reduction (of more than 3 log compared to the free drug) in bacterial counts, which represented a reduction of intracellular bacteria replication of more than 99.9% compared to the untreated infected-cells. To confirm the antimicrobial activity, additional confocal microscopy analyses was performed on infected-cells treated or not with FaPenG NPs, GePenG-SB NPs or free PenG. Using the LIVE/DEAD BacLight bacterial viability assay, it was possible to distinguish between dead bacteria (labelled in red) and living ones (labelled in green). As shown on Fig. 4B, when incubated with free PenG or FaPenG NPs, most of the intracellular bacteria remained still alive, while treatment with GePenG-SB NPs, succeeded to kill most of intracellular bacteria, as soon as 6 h after treatment.

### Intracellular PenG release

**Figure 5:**
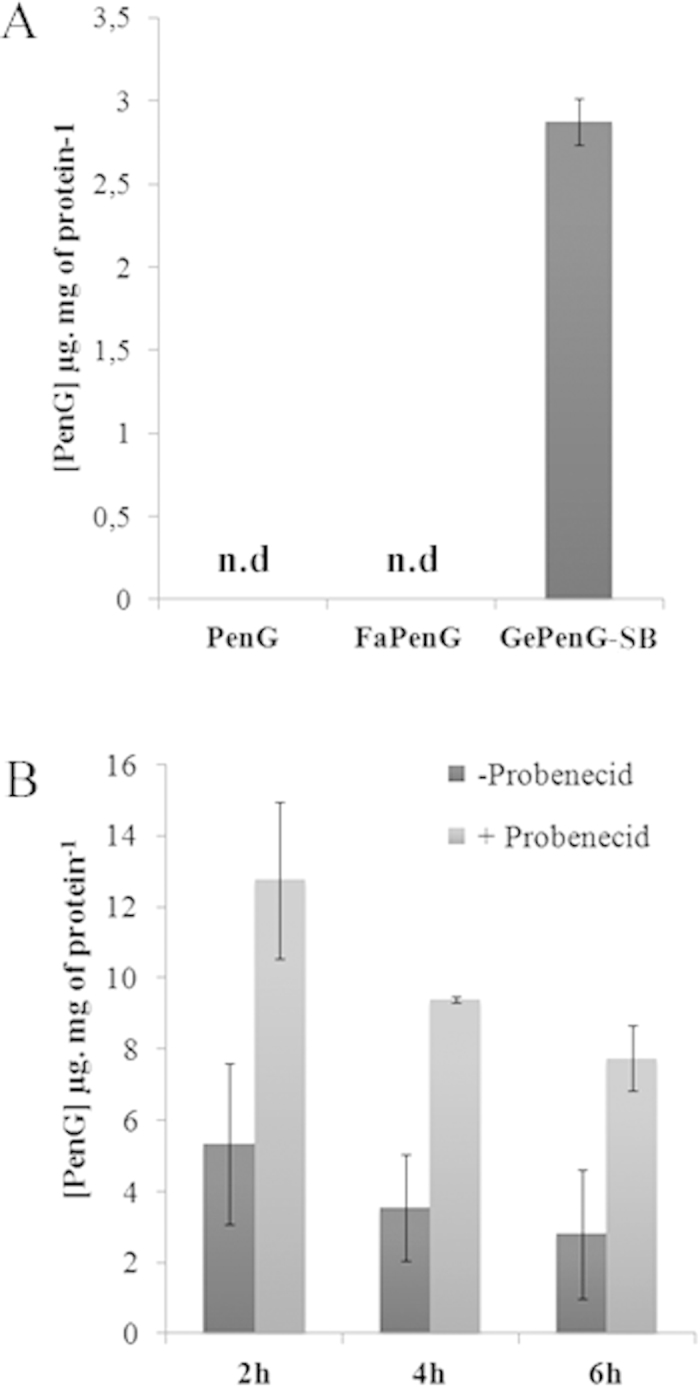
Quantification of intracellular Penicillin G in RAW 264.7 cells. (**A**) After 90 min incubation with free PenG, FaPenG NPs or GePenGpH NPs (at 20 μg.mL^−1^, equiv. PenG), intracellular PenG was estimated using HPLC method; n.d. means: no PenG detected. (**B**) After 2 h, 4 h and 6 h incubation with GePenG-SB NPs (20 μg.mL^−1^, equiv. PenG), in the presence or in the absence of Probenecid (15 mM). Intracellular PenG was estimated using HPLC method.

**Figure 6:**
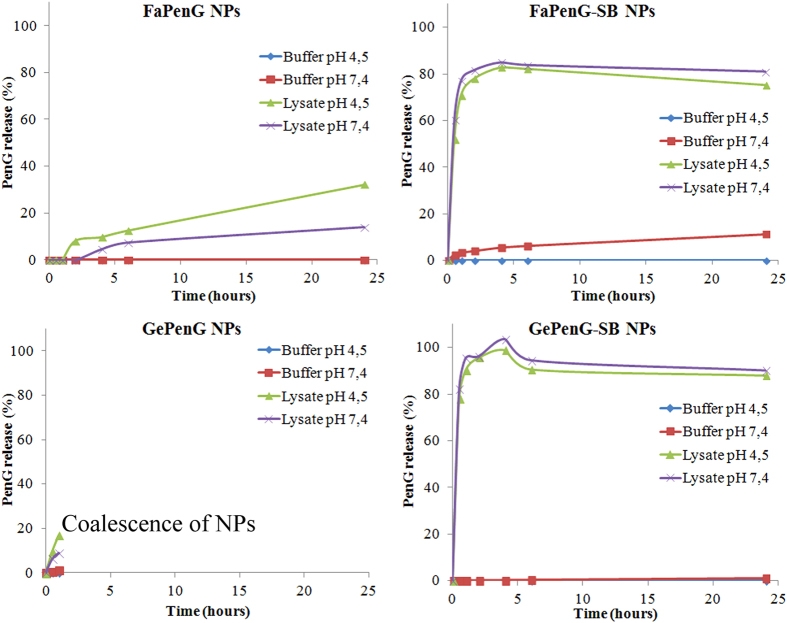
*In vitro* release of PenG from NPs (FaPenG; FaPenG-SB; GePenG; GePenG-SB). NPs (0.1 mg.mL^−1^ equiv. PenG)were incubated at pH 4.5 or 7.4, in the presence or in the absence of cell lysates at 37 °C and PenG release was estimated by HPLC method.

In order to understand the mechanism underlying the better antimicrobial activity of GePenG-SB NPs comparatively to FaPenG NPs, an intracellular penicillin-release pattern has been established by HPLC. So far, most of the studies dealing with nanocarriers for antimicrobial agents have shown drug release profiles into simple buffer such as PBS^28^ or in serum^29^, but never directly into the cells. Therefore, we have developed an original dosage method allowing monitoring and quantification of PenG intracellularly (Supplementary Fig. S7). In this experiment, cells were treated with free PenG, FaPenG NPs or GePenG-SB NPs at concentration of 20 μg.mL^−1^ (equivalent PenG). After 90 min of incubation, cells were washed several times in order to remove all extracellular remaining PenG or NPs and then lysed, in order to extract the intracellular PenG for HPLC measurement. Remarkably, only in those cells treated with GePenG-SB NPs, it was possible to detect the antibiotic up to a concentration of 3 μg PenG.mg-1 of total protein, which represented 2.5% of the total amount of PenG incubated (Fig. 5A). To be noted that, after 90 min of treatment, only about 3% of GePenG-SB NPs were cell internalised (Fig. 3A), meaning that the release of PenG occurred quickly after NPs cell capture. However, after 24 h incubation of the same NPs, no intracellular PenG could be detected which could be explained either by the intracellular degradation of PenG or by its excretion through cell efflux pumps. Therefore, we monitored the stability of PenG over time, in several *in vitro* buffers with pH ranging from 7.4 to 4.5 and it was observed that PenG was rather stable at physiological pH, although degrading at acidic pH as shown on supplementary Figure S7B. Then, we also checked for the second hypothesis using efflux pumps inhibitor. Thus, treated cells were incubated, with probenecid, an efflux pumps inhibitor, usually prescribed with penicillin or other β-lactam antibiotics treatment^30^. Although the co-incubation of GePenG-SB NPs with probenecid significantly increased the intracellular concentration of PenG, in comparison to probenecid free treatment (at 2 h, 4 h and 6 h), it was observed that this value still decreased over time (from 13 μg PenG.mg^−1^ of protein at 2 h to 7.8 μg PenG.mg^−1^ of protein at 6 h), until to become not detectable at 24 h. This suggested that probably, both events occurred: intracellular degradation of PenG that could be release in the lysosomal compartment and excretion through efflux pumps (Fig. 5B). Interestingly, even in the presence of probenecid, the antibiotic has been never detected intracellularly after treatment with either free PenG or with FaPenG NPs, demonstrating once again that PenG cannot be retained into eukaryotic cells^3^ or be efficiently released from the FaPenG NPs (Fig. 5A). This highlights the importance to design nanomedicines with environmentally-sensitive bonds for efficient intracellular antibiotherapy. Owing to the intracellular localisation of nanoparticles (ie. late endosomes and/or lysosomes), we investigated if acidic pH or enzymatic environment (or both) were able to trigger PenG release. Thus, NPs either with ester or acylal linker were incubated at pH 4.5 or 7.4, in the presence or in the absence of cell lysates. Clearly, in the absence of cell extracts and regardless the pH, there was no release of intact PenG from NPs both with or without sensitive-bond (Fig. 6). On the contrary, the presence of cell extracts triggered a fast PenG release, mainly with NPs bearing an acylal sensitive linker (ie., GePenG-SB and FaPenG-SB) (Fig. 6).

## Discussion

In previous reports we explored the remarkable properties of squalene to form nanoparticles when conjugated to different kinds of drugs (i.e. anti-cancer, antiviral and antibiotic)^19^,^23^,^31^. It has been shown that, in general, the resulting nanomedicines displayed better pharmacological activity than the parent drugs^32^,^33^. The aim of the current study was to enlarge this ground-breaking concept to natural terpenes with lower Mw (i.e. geranyl and farnesyl) for antibiotherapy purpose, in order to have available a range of nanocarriers with different functionalities. In this study, four different prodrugs of Penicillin G were synthesised, using farnesyl and geranyl both involved in the cholesterol biosynthesis pathway. Importantly, we added an environemental-responsive linker (-SB) between the antibiotic and the geranyl/farnesyl moieties, in order to trigger drug release in a specific environment such as inside infected-cells.

As our approach was based on the covalent binding between a terpenic moiety and an antibiotic, the use of shorter isoprene chains, automatically increased the drug loading (from 45% for SqPenG-SB NPs^19^ to 54% and 61% for respectively FaPenG-SB NPs and GePenG-SB NPs, see Table 1). As outlined above, high drug loading is an important feature for antibiotherapy as in some intracellular infections, like tuberculosis, high concentrations of so-called “second-line antibiotics” have to be used to treat extensively drug-resistant tuberculosis^34^. Thus, spherical and monodispersed nanoassemblies with average diameter ranging from 195 nm to 260 nm were obtained in water using an easy formulation process (nanoprecipitation/solvent evaporation), except for GePenG NPs which were unstable in size. We suppose that this difference in size and stability could be due to a decrease in the electric surface charge of GePenG bioconjugate and/or in different supersaturation states compared to the other bioconjugates. In fact, for a same concentration, it is expected that at high supersaturation, a growth process called DLCA (diffusion limitated cluster-cluster aggregation) is preferred and this leads to NPs with lower size while at low supersaturation a nucleation and diffusion limited growth mechanism lead to formation of bigger NPs^35^. However, to confirm this hypothesis, further experiments should be done.

One of the requirement of this study was to design NPs with high drug payload but without cytotoxic effect at high concentrations, even if on a conceptual level, the drug release was not bacterial-specific but unspecifically triggered by elements present in the human host. Indeed, we have chosen to use a concentration of 20 μg.mL^−1^ of NPs (equiv PenG) which represents 666 times the MIC of PenG. In this view, FaPenG-SB NPs has proved to be as cytotoxic as squalene-corresponding NPs, on RAW 264.7 cells after 24 h of treatment, likely due to the surface active properties of the farnesylacetic acid, which is released after the hydrolysis of the sensitive linker (Table 3 and Movie M5). The fact that FaPenG NPs were less cytotoxic confirmed this hypothesis, since it was shown that the absence of sensitive-bond on the prodrug did not allow penicillin G release and production of farnesylacetic acid (Tables 2 and 3). But the more interesting observation was that the addition of an acylal sensitive-bond between the isoprene and the antibiotic (ie., GePenG-SB and FaPenG-SB NPs) allowed triggering of antibiotic release, without any influence of the pH (Fig. 6). Moreover, these nanoparticles have displayed similar antimicrobial activity on *Staphylococcus aureus* as free PenG, likely due to the efficient release of this antibiotic, while the NPs bearing a single ester bound (ie., FaPenG) could not (Table 2)^36^. Finally, it was found that the less cytotoxic GePenG-SB nanoparticles were able to decrease the intracellular bacterial count by 99.9% which has been never reached before with this model. The fast and remarkable killing of intracellular *S. aureus* by these NPs was explained by: (i) an important intracellular capture, followed by (ii) a Penicillin G release, triggered by the nanocarrier’s sensitivity to the enzyme-rich intracellular environment. Consequently, we demonstrated the strong antimicrobial potential of this sensitive-bond nanoantibiotic with high drug payload and specific antibiotic delivery in infected intracellular compartments. This approach could be adapted to other antimicrobial agents and opens promising perspectives to combat invasive bacterial infections, which represents a worldwide public health concern and a major burden for the daily industry.

## Materiel and Methods

### Conjugate Synthesis and Characterisation

Geranyl and farnesyl conjugates GePenG (**1**) and FaPenG (**2**) were synthesised by condensation of the sodium salt of PenG in DMF with geranyl and farnesyl bromoacetates in 82% and 68% yield respectively. Geranyl- and farnesylacetic acid chloromethyl esters were obtained from the corresponding acid by treatment with chloromethyl chlorosulfate according to the literature procedure^37^. Subsequent alkylation of the sodium salt of penicillin G with the chloromethyl esters in DMF afforded conjugate **3** and **4** in 74% and 70% yield respectively^38^. Terpenyl conjugates **1**–**4** were found quite stable, surviving chromatographic purification over silica gel and were fully characterized by spectroscopic methods.

### NP preparation and characterization

Nanoparticles were easy prepared by nanoprecipitation. Briefly, 4 mg of GePenG, FaPenG, GePenG-SB or FaPenG-SB bioconjugates were dissolved in 500 μL of ethanol before drop-wise addition, under constant stirring, into 1 mL of sterilized MilliQ water (Fig. S1). Then, ethanol was completely evaporated at 30 °C using a Rotavapor®. For fluorescent labelling of these NPs, the green dye BODIPY® FL C12 cholesteryl ester (Invitrogen) was used and added to the organic phase before nanoprecipitation together with the different prodrugs at 0.5% molar ratio. The average diameter of the nanoparticles, when diluted (1:33) in MilliQ water, was determined at 25 °C by dynamic light scattering (DLS), using a Zetasizer Nano 6.12, (Malvern Instrument Ltd, Worcestershire, UK). The zeta potential (ζ) was measured using the ZetaSizer Nano 6.12, (Malvern Instrument Ltd, Worcestershire, UK) after dilution (1:25) in 1 mM KCl. All measurements were performed at least in triplicate for each formulation.

### Cryogenic Transmission Electron Microscopy

Nanoparticle’s morphology was observed by cryogenic transmission electron microscopy. Briefly, one drop (5 μL) of the nanoassemblies suspension (4 mg.mL^−1^) was deposited onto a perforated carbon film mounted on a 200-mesh electron microscopy grid. Most of the drop was removed with a blotting filter paper and the residual thin films remaining within the holes were quick-frozen by plunging them in liquid ethane cooled with liquid N_2_. The specimen was then transferred using liquid N_2_ to a cryo-specimen holder and observed using a 200 kV JEOL 2100HC electron microscope.

### Bacterial strain and cell line

The experiments herein utilized *Staphylococcus aureus* subsp. *aureus* Rosenbach strain (ATCC 55585) which was obtained from the ATCC and growth in Brain Heart Infusion (BHI) media (Invitrogen) at 37 °C. This strain is penicillin-sensitive.

Mouse leukemic monocyte macrophage cell line (RAW 264.7) was cultured in RPMI 1640 (Roswell Park Memorial Institute) medium supplemented with 10% or 0.5% of inactivated fetal bovine serum (FBSd) (Gibco) at 37 °C in humidified atmosphere containing 5% CO_2_.

### MICs (minimum inhibitory concentrations) determination

Cultures were grown in 10 mL of BHI broth, overnight at 37 °C shaking at 200 rpm. These cultures were then washed and diluted to a final concentration of 5 × 10^5^ bacteria.mL^−1^ in BHI media containing different concentrations of NPs or free penicillin G (ranging from 0 to 225 μg.mL^−1^). After 18 hours incubation at 37 °C and shaking at 200 rpm, bacterial densities were estimated by diluting and plating bacterial on BHI agar followed by incubation overnight at 37 °C. The MIC was the lowest concentration for which there was no bacterial growth (less than 30 CFU).

### Cytotoxicity of NPs on RAW 264.7 cells

Cytotoxicity of nanoparticles on macrophages was evaluated by the MTT (3-(4,5-dimethylthiazol-2-yl)-2,5-diphenyltetrazolium bromide) method^39^. RAW 264.7 cells were seeded in 96-well plates (at 5 × 10^3^ cells per well) and incubated in RPMI medium containing 10% FBSd for 24 h. The cells were then incubated during 24 h at 37 °C with serial concentration of NPs in RPMI medium containing 0.5% FBSd. Afterward, cells were washed and MTT solutions were added at final concentration of 0.5 mg.mL^−1^. After 2 h at 37 °C, medium was removed and 100 μL of DMSO was added, mixed carefully and kept in dark at room temperature for 10 min. Absorbance was measured at 570 nm using a plate reader (Metertech Σ960, Fisher Bioblock, Illkirch, France). The percentage of surviving cells was calculated as the ratio of treated to untreated cells. The inhibitory concentration 50% (IC_50_) of the treatments was determined from the dose–response curve. All experiments were set up in triplicate.

### Cellular uptake of NPs

#### Kinetics of cell internalisation by FACS

2 × 10^5^ RAW 264.7 cells were seeded into 24-wells plates and incubated in RPMI medium containing 10% FBSd at 37 °C, 5% CO_2_. After 18 hours, cells were washed with PBS and fresh RPMI medium containing 0.5% FBSd and fluorescent NPs (25 μg.mL^−1^ of either GePenG-SB or FaPenG) was added. After exposure to cells for required time (30 min, 1, 2, 4, 6 and 24 h at 37 °C), the NPs suspension was removed and the cells were washed three times with PBS and treated with 0.25% trypsin for 10 min at 37 °C. Finally, cells were resuspended directly with PBS buffer for cytometry measurements (FACS). Cell suspensions were analyzed by flow cytometry (Accuri C6, BD Biosciences), and the mean fluorescence intensities were collected on channel FL-1. Results were expressed as the ratio of the mean fluorescence intensity of each sample to the mean fluorescence intensity of non-treated cells. All measurements were performed in triplicate or more.

### Confocal microscopy

For time-lapse confocal imaging, cells were plated on 6-wells plates with 25 mm diameter glass coverslips, at cell densities of 5 × 10^5^ cells by well, and treated as described for flow cytometry sample preparation. For imaging, cells incubated with fluorescent NPs were placed into Attofluor® Cell Chamber (Life Technology) and maintained at 37 °C using a stage heater. The cells were observed using an inverted confocal laser scanning microscope LSM 510-Meta (Carl Zeiss, Germany) using a Plan-Apochromat 63X/1.4 objective lens, equipped with an argon laser (488nm excitation wavelength). Green fluorescence was collected with a 505–550 nm band-pass. The pinhole was set at 1.0 Airy unit. 12 bit numerical images were acquired with LSM 510 software version 3.2.

### Intracellular antimicrobial activity of NPs

#### CFU determination

RAW 264.7 cells were seeded in 24-well plates in RPMI with 10% FBSd at a concentration of 1.5 × 10^5^ cells per well and allowed to adhere overnight. The medium was then removed from the wells, and the cells were washed with phosphate-buffered saline (PBS). An inoculum of 2 × 10^6^ CFU of *Staphylococcus aureus* was added to each well. After 2 h, the culture plates were washed three times with PBS and incubated with fresh medium containing 50 μg.ml^−1^ gentamicin, for 90 min at 37 °C, in order to kill remaining extracellular bacteria. The cells were then washed three additional times with PBS and then treated with NPs or free PenG (20 μg.mL^−1^ equiv. PenG) in RPMI supplemented with 0.5% FBSd during requiring time. Then, cells were washed with cold PBS three-times before the lysis of the cells with ice-cold sterilized MilliQ water in order to release intracellular bacteria. The lysates containing released bacteria were serially diluted (1:10 in 0.9% NaCl), plated on BHI agar plates, and cultured overnight at 37 °C. The limit of detection for this plate counting method was 200 CFU.ml^−1^.

#### Confocal microscopy

To distinguish vivid bacteria with intact cell walls from dead bacteria with compromised membranes, the LIVE/DEAD BacLight Bacterial Viability kit (Molecular Probes) was used^40^. RAW 264.7 cells were infected by *S. aureus*, and then treated in similar conditions than the ones explained above for CFU determination. Thereafter, the cells were washed three-times with PBS, fixed with 1% PFA, and permeabilized with 0.2% Triton-X100. They were then brought into the mix of the LIVE/DEAD BacLight bacterial viability kit for 15 min in the dark. After washing with PBS, RAW 264.7 cells were observed with an inverted confocal laser scanning microscope LSM 510-Meta (Carl Zeiss, Germany) using a Plan-Apochromat 63X/1.4 objective lens, equipped with an argon (488 nm excitation wavelength) and a helium neon laser (543 nm excitation wavelength). The green and red fluorescence were collected with a 505–550 nm band-pass and a 560 nm long pass emission filter respectively, under a sequential mode. The pinhole was set at 1.0 Airy unit. 12 bit numerical images were acquired with LSM 510 software version 3.2.

### *In vitro* release of Penicillin G

#### Chromatographic and apparatus conditions

The chromatography system used was composed by reverse-SBase HPLC (Waters) with a C18 column. The system consisted of a Waters 1525 Binary LC pump, a Waters 2707 Autosampler, a C18 Yptisphere column (5 μm, Interchim), HPLC column temperature controllers (model 7950 column heater and chiller; Jones Chromatography) and a Waters 484 programmable fluorescence detector. The mobile phase was composed of acetonitrile/trifluoroacetic acid [40/0.1% (v/v)], and the flow rate was 1 mL.min^−1^. The column temperature was maintained at 25 °C, the UV–vis detection wavelength was set at 210 nm, and the injection volume was 10 μL. In these conditions, the retention time of PenG was 3.8 min (Supplementary Fig. S8).

### Sample preparation for intracellular PenG release

RAW 264.7 cells were seeded on Petri dishes (diameter 10 cm) in RPMI supplemented with 10% FBSd and allowed to adhere overnight. The cells were washed with phosphate-buffered saline (PBS) and NPs or free PenG diluted in RMPI + 0.5% FBSd (20 μg.mL^−1^ equiv. PenG) were added during 90 min, 2 h, 4 h, 6 h, 24 h. The treated-cells were washed three times with cold PBS and lysed in cold Lysis Buffer (Pierce) for 5 min and then centrifuged at 11,000 × g for 45 min at 4 °C. The supernatant was collected and dried but before, a small aliquot was kept in order to determine the protein concentration using BCA assay (BioRad). The dry residue was reconstituted in 200 μL of the mobile phase, centrifuged at 5000 × g for 10 min and then 10 μL of the sample was injected into the HPLC for analysis.

### PenG release in buffers or in cell lysates

Drug release from NPs was determined in PBS at pH 7.4 and in sodium citrate 0.1M pH 4.5. NPs suspensions (0.1 mg.mL^−1^ equiv. PenG) were placed in several 1.5 mL tubes and incubated at 37 °C on a wheel. At predetermined time intervals, an aliquot was analysed by HPLC following the process described above.The same set of experiment was performed with 1mg of RAW cell lysates (prepared as described above) at pH adjusted at 7.4 and 4.5.

## Additional Information

**How to cite this article**: Abed, N. *et al.* An efficient system for intracellular delivery of beta-lactam antibiotics to overcome bacterial resistance. *Sci. Rep.* **5**, 13500; doi: 10.1038/srep13500 (2015).

## Supplementary Material

Supporting information

Supplementary Movie M1

Supplementary Movie M2

Supplementary Movie M3

Supplementary Movie M4

Supplementary Movie M5

## Figures and Tables

**Table 1:** Physicochemical characterisation of nanoparticles^(a)^.

Nanoparticles	Size [nm]	Polydispersty index	Zeta Potential [mV]	Drug payload [%]^(b)^
FaPenG	259 ± 18	0.08 ± 0.04	−47 ± 15.1	56
FaPenG-SB	195 ± 3	0.04 ± 0.01	−61.5 ± 3.3	54.7
GePenG	464 ± 62	0.09 ± 0.04	−20.6 ± 6	63.2
GePenG-SB	250 ± 22	0.07 ± 0.04	−63.5 ± 4.8	61.5
FaPenG::BC	254 ± 19	0.09 ± 0.02	−35.5 ± 6.7	—
FaPenG-SB::BC	198 ± 9	0.06 ± 0.02	−60.5 ± 4.5	—
GePenG-SB::BC	280 ± 17	0.05 ± 0.03	−62 ± 1	—

Notes: ^(a)^Each value represents the average of more than three different experiments ± standard deviation.

^(b)^Drug payload = (Mw_PenG_ × 100)/Mw_NPs_.

**Table 2:** Minimum Inhibitory Concentration (MIC) of nanoparticles on *Staphylococcus aureus* ATCC 55585, a penicillin-sensitive strain.

Compounds	MIC (μg.mL^−1^)	MIC equivalent PenG (μg.mL^−1^)
Penicillin G	0.03	0.03
NPs FaPenG	>225	>126
NPs FaPenG-SB	0.156	0.085
NPs GePenG-SB	0.1	0.06
NPs FaCH_2_CO_2_H	>225	—
NPs GeCH_2_CO_2_H	>225	—

**Table 3:** NPs (i.e. GePenG-SB, FaPenG, FaPen-SB and SqPenG-SB) and PenG cytotoxicity (IC_50_) on RAW 264.7 cell after 24 h treatment in RPMI supplemented with 0.5% FBSd at 37 °C.

Nanoparticles	SqPenG-SB	FaPenG-SB	GePenG-SB	FaPenG	PenG
IC_50_ value (μg.mL^−1^)	18 ± 2.88	22 ± 10.25	72.5 ± 10.40	112 ± 17.77	>100

Note: IC_50_ represent the drug concentration required to inhibit the cell viability by 50%

Each value represents the average three independent experiments ± standard deviation
