# Supplementary material for: An efficient system for intracellular delivery of beta-lactam antibiotics to overcome bacterial resistance
Source: Sci Rep. 2015 Aug 27;5:13500. doi: 10.1038/srep13500 (PMC4550931; doi:10.1038/srep13500)
Supplement: Supporting information [file srep13500-s1.pdf]

Supplementary information

An efficient system for intracellular delivery of beta-lactam antibiotics to overcome bacterial resistance.

Nadia Abed[1], Fatouma Saïd-Hassane[1], Fatima Zouhiri[1], Julie Mougin[1], Valérie Nicolas[2], Didier Desmaële[1], Ruxandra Gref[1] and Patrick Couvreur[1]*

[1]Faculty of Pharmacy, Institut Galien UMR CNRS 8612, University of Paris-Sud XI, 5 Rue Jean-Baptiste Clément, 92296 Châtenay-Malabry Cedex, France

[2]Faculty of Pharmacy, INSERM IFR 141 University of Paris-Sud XI, 5 Rue Jean-Baptiste Clément, 92296 Châtenay-Malabry Cedex, France

*To whom correspondence should be addressed: Pr. Patrick Couvreur

Université Paris-Sud, Faculté de Pharmacie, CNRS-UMR 8612, Institut Galien Paris-Sud,

5, rue J.B. Clément, 92296 Châtenay-Malabry, Cedex, France.

patrick.couvreur@u-psud.fr

Tel: +33 (0) 1 46 83 53 96

Fax: +33 (0) 1 46 83 59 46.

Supplementary Methods section:

<u>*Synthesis of different bioconjugates:*</u>

2-[(2S,5R,6R)-3,3-Dimethyl-7-oxo-6-(2-SBenylacetamido)-4-thia-1-azabicyclo[3.2.0]heptane-2-carbonyloxy]acetic Acid (2*E*)-3,7-Dimethylocta-2,6-dien-1-yl Ester (1): A solution of Penicillin G sodium salt (400 mg, 1.12 mmol) and geranyl bromoacetate (779 mg, 2.84 mmol) in dry DMF (15 mL) was stirred at room temperature for 72 h. The reaction mixture was then concentrated *in vacuo* and the residue was purified by flash column chromatography on silica gel (hexane/AcOEt, 4:1) to afford the title compound as a colourless wax (485 mg, 82%). $[\alpha]_D^{21}$ +3.3 (*c* = 4.14, $CHCl_3$); IR (neat, cm^{-1}) ν: 3400-3100, 2665, 2931, 2854, 1785, 1750, 1691, 1661, 1496, 1455, 1394, 1374, 1290, 1197, 1174, 1152; ^{1}H NMR (300 MHz, $CDCl_3$) δ 7.45-7.25 (m, 5 H, Ph*H*), 6.08 (d, *J* = 8.9 Hz, 1 H, CON*H*), 5.67 (dd, *J* = 9.3, 4.3 Hz, 1 H, H-6), 5.50 (d, *J* = 4.2 Hz, 1 H, H-5), 5.32 (t, *J* = 6.4 Hz, 1 H,, $OCH_2CH{=}C(CH_3)$), 5.07 (m, 1H, $CH_2CH{=}C(CH_3)_2$), 4.78 (d, *J* = 15.8 Hz, 1 H, OCH_2CO_2), 4.68 (d, *J* = 7.2 Hz, 2 H, $OCH_2CH{=}C(CH_3)$), 4.59 (d, *J* = 15.8 Hz, 1 H, OCH_2CO_2), 4.43 (s, 1 H, H-2), 4.13 (t, *J* = 6.7 Hz, 2 H, $CO_2CH_2CH_2$), 3.61 (s, 2 H, $PhCH_2CO$), 2.16-2.00 (m, 4 H, $CH_2CH_2CH{=}C(CH_3)_2$), 170 (s, 3 H), 1.68 (s, 3 H), 1.60 (s, 3H), 1.57 (s, 3 H, $SC(CH_3)_2$), 1.50 (s, 3 H, $SC(CH_3)_2$); ^{13}C NMR (75 MHz, $CDCl_3$) δ: 173.6 (C, C-7), 170.3 (C, CO_2), 166.9 (C, *C*O), 166.8 (C, *C*O), 143.3 (C, $OCH_2CH{=}C(CH_3)$), 133.7 (C, Ph), 131.8 (C, $CH_2CH{=}C(CH_3)_2$), 129.4 (2 CH, Ph), 129.0 (2 CH, Ph), 127.5 (CH, Ph), 123.4 (CH, $CH_2CH{=}C(CH_3)_2$), 117.2 (CH, $OCH_2CH{=}C(CH_3)$), 70.1 (CH, C-3), 67.8 (CH, C-5), 64.5 (C, C-2), 62.3 (CH_2 $OCH_2CH{=}C(CH_3)$, 61.2 (CH_2, OCH_2CO_2), 58.4 (CH, C-6), 43.2 (CH_2, $PhCH_2CO$), 39.4 (CH_2, $OCH_2CH{=}C(CH_3)CH_2$), 31.0 (CH_3, $SC(CH_3)_2$), 26.6 (CH_3, $SC(CH_3)_2$), 26.1 (CH_2, $OCH_2CH{=}C(CH_3)CH_2CH_2$), 25.6 ($CH_3$), 17.6 ($CH_3$), 16.4 ($CH_3$); MS

(+APCI): m/z (%) = 529.2 (9) $[M+H]^+$, 354 (100), $[M-PhCH_2CONHCH=C=O]^+$, 218 (66); HRMS (+ESI) calcd for $C_{28}H_{36}N_2O_6SNa$: 551.2186, found 551.2164; Anal. calcd for $C_{28}H_{36}N_2O_6S$, C 63.61, H 6.86, N 5.30; found: C 63.58, H 6.88, N 5.19.

2-[(E,E)-(2S,5R,6R)-3,3-dimethyl-7-oxo-6-(2-SBenylacetamido)-4-thia-1-azabicyclo[3.2.0]heptane-2-carbonyloxy]acetic Acid (2E,6E)-3,7,11-Trimethyldodeca-2,6,10-trien-1-yl Ester(2): A solution of Penicillin G sodium salt (197 mg, 054 mmol) and (2*E*,6*E*)-3,7,11-trimethyldodeca-2,6,10-trien-1-yl 2'-bromoacetate (125 mg, 0.36 mmol) in dry DMF (3 mL) was stirred at room temperature for 40 h. The reaction mixture was then concentrated *in vacuo* and the residue was purified by flash column chromatography on silica gel (cyclohexane/AcOEt, 6:1) to afford the title compound as a yellow oil (182 mg, 85%). $[\alpha]_D^{20}$ +1.0 (c = 1.46, $CHCl_3$); IR (neat, cm^{-1}): ν = 2980 – 2900, 1789, 1751, 1693, 1659, 1535, 1495, 1453, 1394, 1354, 1291, 1198, 1176, 1151, 1130, 770, 731; ^1H NMR (400 MHz, $CDCl_3$) δ 7.36 – 7.24 (5 H, m, Ph*H*), 6.32 (1 H, d, *J* = 7.6 Hz, CON*H*), 5.64 (1 H, dd, *J* = 8.9 Hz, *J* = 4.2 Hz, H-6), 5.49 (1 H, d, *J* = 4.2 Hz, H-5), 5.32 (1 H, t, *J* = 7.1 Hz, $OCH_2CH=C(CH_3)$), 5.08 (2 H, t, *J* = 6.0 Hz, H-6’, H-10’), 4.75 (1 H, d, *J* = 15.8 Hz, OCH_2CO_2), 4.67 (2 H, d, *J* = 7.2 Hz, $OCH_2CH=C(CH_3)$), 4.57 (1 H, d, *J* = 15.8 Hz, OCH_2CO_2), 4.42 (1 H, s, H-2), 3.61 (2 H, s, $PhCH_2CO$), 2.12 – 1.94 (8 H, m, H-4’, H-5’, H-8’, H-9’), 1.69 (3 H, s, $OCH_2CH=C(CH_3)$), 1.66 (3 H, s, CH_3), 1.58 (6 H, s, $2CH_3$), 1.55 (3 H, s, $SC(CH_3)_2$), 1.50 (3 H, s, $SC(CH_3)_2$); ^{13}C NMR (75 MHz, $CDCl_3$) δ 173.7 (C, C-7), 170.4 (C, NH*C*=O), 167.0 (C, CO_2), 166.8 (C, CO_2), 143.6 (C, $OCH_2CH=C(CH_3)$), 135.5 (C, $HC=C(CH_3)$), 133.9 (C, Ph), 131.3 (C, $HC=C(CH_3)_2$), 129.5 (2CH, Ph), 129.0 (2CH, Ph), 127.5 (CH, Ph), 124.3 (CH, $CH=C(CH_3)$), 123.5 (CH, $CH=C(CH_3)$), 117.3 (CH, $OCH_2CH=C(CH_3)$), 70.2 (CH, C-3), 67.9 (CH, *C*-5), 64.6 (C, C-2), 62.4 (CH_2, $OCH_2CH=C(CH_3)$), 61.3 (CH_2, OCH_2CO_2), 58.6 (CH, C-6), 43.2 (CH_2, $PhCH_2CO$), 39.6

(CH_2, CH=C(CH_3)*C*H_2), 39.5 (CH_2, CH=C(CH_3)*C*H_2), 31.1 (CH_3, SC(C*H*$_3$)$_2$), 26.7 (CH_3, SC(C*H*$_3$)$_2$), 26.6 (CH_2, CH=C(CH_3)CH_2*C*H_2), 26.1 (CH_2, CH=C(CH_3)CH_2*C*H_2), 25.7 (CH_3) 17.7 (CH_3) 16.5 (CH_3) 16.0 (CH_3); MS (APCI$^-$): *m/z* (%) = 595 (100) [M-H]$^-$; HRMS (+ESI) calcd for $C_{33}H_{44}N_2O_6SNa$: 619.2789, found 619.2812; Anal. calcd for $C_{33}H_{44}N_2O_6S$, C 66.42, H 7.43, N 4.69; found: C 66.27, H 7.80, N 4.48.

***(E)*-5,9-Dimethyldeca-4,8-dienoic Acid Chloromethyl Ester**: 1.5 M K_2CO_3 aq. solution (2 mL, 3 mmol) and chloromethyl chlorosulfate (0.12 mL, 1.1 mmol) were sequentially added to a solution of (*E*)-5,9-dimethyldeca-4,8-dienoic acid (197 mg, 1 mmol) and *n*-Bu_4NHSO_4 (37 mg, 0.1 mmol) in CH_2Cl_2 (2 mL). The reaction mixture was vigorously stirred at room temperature for 1 h and CH_2Cl_2 (10 mL) was added. The organic layer was separated, washed with brine (2 × 10 mL), dried over anhydrous $MgSO_4$, filtered and concentrated *in vacuo* to afford the title compound as a pale yellow oil (244 mg, quant.) which was used into the following step without any further purification. IR (neat, cm^{-1}): ν = 2961 – 2860, 1791, 1770, 1763, 1439, 1376, 1333, 1262, 1174, 1123, 1121, 1049, 998, 725, 717, 696; ^{1}H NMR (300 MHz, $CDCl_3$) δ 5.67 (2 H, s, OC*H*$_2$Cl), 5.07 (2 H, m, H-4, H-8), 2.45 – 2.23 (4 H, m, O_2CC*H*$_2$C*H*$_2$CH=), 2.08 – 1.90 (4 H, m, =C(CH_3)C*H*$_2$C*H*$_2$CH=), 1.65 (3 H, s, CH_3), 1.60 (3 H, s, CH_3), 1.57 (3 H, s, CH_3); ^{13}C NMR (75 MHz, $CDCl_3$) δ 169.2 (C, *C*O_2), 137.0 (C, CH=*C*(CH_3)), 131.5 (C, CH=*C*(CH_3)), 124.2 (CH, *C*H=C(CH_3)), 121.5 (CH, *C*H=C(CH_3)), 65.7 (CH_2, O*C*H_2Cl), 39.7 (CH_2, =C(CH_3)C*H*$_2$C*H*$_2$CH=), 34.3 (CH_2, O_2C*C*$H_2$$CH_2$), 26.7 ($CH_2$, *C*$H_2$HC=C($CH_3$)$_2$), 25.7 ($CH_3$, HC=C(*C*$H_3$)$_2$), 23.3 ($CH_2$, O_2CCH_2*C*H_2), 17.7 (CH_3), 16.0 (CH_3); MS (ESI$^+$): *m/z* (%) = 245 (100) [M+H]$^+$, 242 (40). 179 (18) [M-OCH_2Cl]$^+$

(4*E*,8*E*)-5,9,13-Trimethyltetradeca-4,8,12-trienoic Acid Chloromethyl Ester: 1.5 M K_2CO_3 aq. solution (2 mL, 3 mmol) and chloromethyl chlorosulfate (0.12 mL, 1.1 mmol) were sequentially added to a solution of (4*E*,8*E*)-5,9,13-trimethyltetradeca-4,8,12-trienoic

acid (264 mg, 1 mmol) and *n*-Bu_4NHSO_4 (37 mg, 0.1 mmol) in CH_2Cl_2 (2 mL). The reaction mixture was vigorously stirred at room temperature for 1 h and CH_2Cl_2 (10 mL) was added. The organic layer was separated, washed with brine (2 × 10 mL), dried over anhydrous $MgSO_4$, filtered and concentrated *in vacuo* to afford the title compound as a pale yellow oil (310 mg, quant.) which was used into the following step without any further purification. IR (neat, cm^{-1}): ν = 3000 – 2800, 1769, 1440, 1417, 1384, 1376, 1335, 1260, 1122, 1047, 1015, 998; ^{1}H NMR (300 MHz, $CDCl_3$) δ 5.68 (2 H, s, OC*H*$_2$Cl), 5.17 – 5.04 (3 H, m, *H*C=C(CH_3)), 2.45 – 2.35 (4 H, m, O_2CC*H*$_2$C*H*$_2$CH=), 2.12 – 1.97 (8 H, m, =C(CH_3)C*H*$_2$C*H*$_2$CH=), 1.67 (3 H, s, CH_3), 1.62 (3 H, s, CH_3), 1.59 (6 H, m, CH_3); ^{13}C NMR (75 MHz, $CDCl_3$) δ 171.3 (C, *C*O), 137.2 (C, CH=*C*(CH_3)), 135.1 (C, CH=*C*(CH_3)), 131.2 (C, CH=*C*(CH_3)), 124.4 (CH, *C*H=C(CH_3)), 124.0 (CH, *C*H=C(CH_3)), 121.6 (CH, *C*H=C(CH_3)), 68.5 (CH_2, O*C*H$_2$Cl), 39.7 (CH_2, =C(CH_3)C*H*$_2$C*H*$_2$CH=), 39.6 (CH_2, =C(CH_3)C*H*$_2$C*H*$_2$CH=), 34.1 (CH_2, =C(CH_3)C*H*$_2$C*H*$_2$CH=), 26.7 (CH_2), 26.4(CH_2),25.6 (CH_3 CH=C(*C*H$_3$)$_2$), 23.1 (CH_2), 23.4 (CH_3), 17.6 (CH_3), 15.9 (2 CH_3); MS (ESI$^+$): *m/z* (%) = 313 (100) $[M+H]^+$, 265 (4) $[M–CHCl+H]^+$, 242 (5).

(4*E*)-5,9-Dimethyldeca-4,8-dienoic Acid [(E)-(2S,5R,6R)-3,3-dimethyl-7-oxo-6-(2-SBenylacetamido)-4-thia-1-azabicyclo[3.2.0]heptane-2-carbonyloxy]methyl Ester (3): A solution of Penicillin G sodium salt (186 mg, 0.5 mmol) and (*E*)-chloromethyl 5,9-dimethyldeca-4,8-dienoate (125 mg, 0.51 mmol) in dry DMF (4 mL) was stirred at room temperature for 3 days. The reaction mixture was then concentrated *in vacuo* and the residue was purified by flash column chromatography on silica gel (cyclohexane/AcOEt, 5:1) to afford the title compound as a yellow oil (160 mg, 59%). $[\alpha]_D^{25}$ +1.12 (*c* = 1, $CHCl_3$); IR (neat, cm^{-1}): ν = 3400-3200, 3060 – 2850, 1789, 1661, 1509, 1496, 1454, 1374, 1291, 1198, 1157, 1130, 1116, 1030, 986, 970; ^{1}H NMR (400 MHz, $CDCl_3$) δ 7.36 – 7.22 (5 H, m, Ph),

6.21 (1 H, d, *J* = 8.9 Hz, CON*H*), 5.80 (1 H, d, *J* = 5.6 Hz, OCH_2O), 5.74 (1 H, d, *J* = 5.6 Hz, OCH_2O), 5.63 (1 H, dd, *J* = 8.9 Hz, *J* =4.2 Hz, H-6), 5.48 (1 H, d, *J* =4.2 Hz, H-5), 5.10 – 5.02 (2 H, m, HC=C(CH_3)), 4.37 (1 H, s, H-2), 3.61 (2 H, s, PhCH_2CO), 2.40–2.24 (2 H, m, O_2CCH_2CH_2), 2.09–1.91 (2 H, m, =C(CH_3)CH_2CH_2CH=), 1.66 (3 H, s, CH_3), 1.59 (3 H, s, CH_3), 1.58 (3 H, s, CH_3), 1.43 (3 H, s, SC(CH_3)$_2$), 1.42 (3 H, s, SC(CH_3)$_2$); ^{13}C NMR (100 MHz, $CDCl_3$) δ 173.4 (C, C-7) 171.6 (C, O_2*C*CH_2), 170.3 (NH*C*=O), 166.3 (C, OCH_2O*C*O), 137.2 (C, *C*5'), 133.8 (C, Ph), 131.4 (C, *C*9'), 129.5 (2CH, Ph), 129.0 (2CH, Ph), 127.5 (CH, Ph), 124.0 (CH, *C*-8'), 121.5 (CH, *C*-4'), 79.5 (O*C*H_2O), 69.8 (CH, C-3), 68.0 (CH, C-5), 64.4 (C, C-2), 58.8 (CH, C-6), 43.2 (CH_2, Ph*C*H_2CO), 39.5 (CH_2, =C(CH_3)*C*H_2*C*H_2CH=), 33.9 (CH_2, O_2C*C*H_2), 31.6 (CH_3, SC(CH_3)$_2$), 26.6 (CH_3, SC(CH_3)$_2$), 26.5 (CH_2, CH_2C=C(CH_3)$_2$), 25.6 (CH_3, C=C(*C*H_3)$_2$), 23.0 (CH_2, O_2CCH_2*C*H_2), 17.6 (CH_3, C=C(*C*H_3)$_2$), 15.9 (CH_3, =C(*C*H_3)CH_2CH_2CH=); MS (ESI$^+$): *m/z* (%) = 565 $[M+Na]^+$ (100); HRMS (+ESI) calcd for $C_{29}H_{38}N_2O_6S$,Na: 565.23486, found 565.23180; Anal. calcd for $C_{29}H_{38}N_2O_6S$, C 64.18, H 7.06, N 5.16; found: C 64.79, H 7.49, N 4.71.

(4*E*,8*E*)-5,9,13-Trimethyltetradeca-4,8,12-trienoic Acid [(2S,5R,6R)-3,3-Dimethyl-7-oxo-6-(2-SBenylacetamido)-4-thia-1-azabicyclo[3.2.0]heptane-2-carbonyloxy]methyl Ester (4): A solution of Penicillin G sodium salt (180 mg, 0.5 mmol) and (4*E*,8*E*)-chloromethyl 5,9,13-trimethyltetradeca-4,8,12-trienoate (160 mg, 0.51 mmol) in dry DMF (4 mL) was stirred at room temperature for 3 days. The reaction mixture was then concentrated *in vacuo* and the residue was purified by flash column chromatography on silica gel (cyclohexane/AcOEt, 4:1) to afford the title compound as a yellow oil (216 mg, 70%). $[\alpha]_D^{17}$ +0.98 (*c* = 1, $CHCl_3$); IR (neat, cm^{-1}): ν = 3400-3200, 2960–2920, 1790, 1764, 1689, 1659, 1496, 1455, 1374, 1292, 1240, 1199, 1158, 1131, 1117, 987, 973; ^{1}H NMR (400 MHz, d_6-acetone) δ 7.73 (1 H, br d, *J* = 8.6 Hz, CON*H*), 7.42 – 7.22 (5 H, m, Ph*H*), 5.88 (1 H, d, *J* = 5.6 Hz, OCH_2O), 5.80 (1 H, d, *J* = 5.6 Hz, OCH_2O), 5.65 (1 H, dd, *J* = 9.0 Hz, *J* = 4.2 Hz, H-

6), 5.55 (1 H, d, *J* = 4.2 Hz, H-5), 5.20-5.08 (3 H, m, *H*C=C), 4.39 (1 H, s, H-3), 3.66 (H, d, *J* = 14.6 Hz, PhCH_2CO), 3.61 (H, d, *J* = 14.6 Hz, PhCH_2CO), 2.45–2.25 (4 H, m, O_2CCH_2CH_2), 2.14–1.94 (6 H, m, =C(CH_3)CH_2CH_2CH=), 1.68 (3H s, CH_3), 1.63 (3H s, CH_3), 1.61 (3H s, CH_3), 1.59 (3H s, CH_3), 1.58 (3 H, s, SC(CH_3)$_2$), 1.48 (3 H, s, SC(CH_3)$_2$); ^{13}C NMR (75MHz, CDCl3) δ 173.5 (C, C-7), 171.8 (C, O_2*C*CH_2), 170.5 (C, NH*C*=O), 166.5 (C, OCH_2O*C*O), 137.4 (C, *C*-5'), 135.3 (C, Ph), 133.9 (C, *C*-9'), 131.4 (C, *C*-13'), 129.7 (2CH, Ph), 129.2 (2CH, Ph), 127.8 (CH, Ph), 124.4 (CH, *C*-8'), 124.0 (CH, *C*-12'), 121.7 (CH, *C*-4'), 79.7 (CH_2, O*C*H_2O), 70.0 (CH, *C*-2), 68.2 (CH, *C*-5), 64.6 (C, *C*-3), 59.0 (CH, *C*-6), 43.4 (CH_2, Ph*C*H_2CO), 39.8 (CH_2, *C*6'H_2), 39.7 (CH_2, *C*10'H_2), 34.1 (CH_2, *C*2'H_2), 31.8, (CH_3, SC(CH_3)$_2$), 26.8 (CH_2, *C*7'H_2 or *C*11'H_2), 26.7 (CH_3, SC(CH_3)$_2$), 26.6 (CH_2, *C*7'H_2 or *C*11'H_2), 25.8 (CH_3, C13'*C*H_3), 23.2 (CH_2, *C*3'H_2), 17.8 (CH_3, C13'*C*H_3), 16.1 (CH_3, C5'*C*H_3); MS (ESI$^-$): *m/z* (%) = 609 (100) [M-H]$^-$; HRMS (+ESI) calcd for $C_{34}H_{46}N_2O_6SNa$: 633.2968, found 633.2939; Anal. calcd for $C_{349}H_{46}N_2O_6S$, C 66.86, H 7.59, N 4.59; found: C 66.27, H 7.80, N 4.48.

Intracellular localisation of NPs by confocal microscopy analysis

RAW 264.7 cells were plated on glass coverslips (2×10^5 cells.ml^{-1}) in 24-well tissue-culture plates overnight. Then medium was removed and cells were treated with 20 µg.ml^{-1} of fluorescent NPs (FaPenG::BC or GePenG-SB::BC) in RPMI supplemented with 0.5% FBSd. After 2h of incubation at 37°C, 5% CO_2, cells were washed 3-times with PBS and the coverslips were incubated with 3% PFA-PBS for 15 min at room temperature. Cells were washed 3-times with PBS and 2-times with PBS-BSA 1% and then incubated with APC conjugated anti- CD11b (Invitrogen, 1:500) in PBS-BSA 1% for 30 min at 37°C. Finally, cells were washed 3-times with PBS and mounted with Dako anti-fading reagent (Dako), and examined with an inverted Zeiss LSM-510 META confocal laser scanning microscope.

Disk diffusion assay (MIC)

Staphylococcus aureus susceptibility to PenG or PenG-loaded NPs were assessed by a disk diffusion assay using 6-mm filter paper disks (Bio-Rad) as recommended by the European Committee on Antimicrobial Susceptibility Testing. From overnight cultures, bacterial suspensions were prepared at the 0.5 McFarland turbidimetric standards. Then, the suspensions were poured over a Mueller-Hinton (MH) agar plate and after drying, 6 mm disks containing the free PenG or PenG-loaded NPs (at 10 µg.mL^{-1} equiv. PenG) were placed on the agar surface. After overnight incubation at 37°C we determined the diameter of the growth inhibition zone that reflects the strain sensibility to the free antibiotic/ antibiotic-loaded NPs. Two experiments were carried out separately with different formulations.

Supplementary Figures and Movies

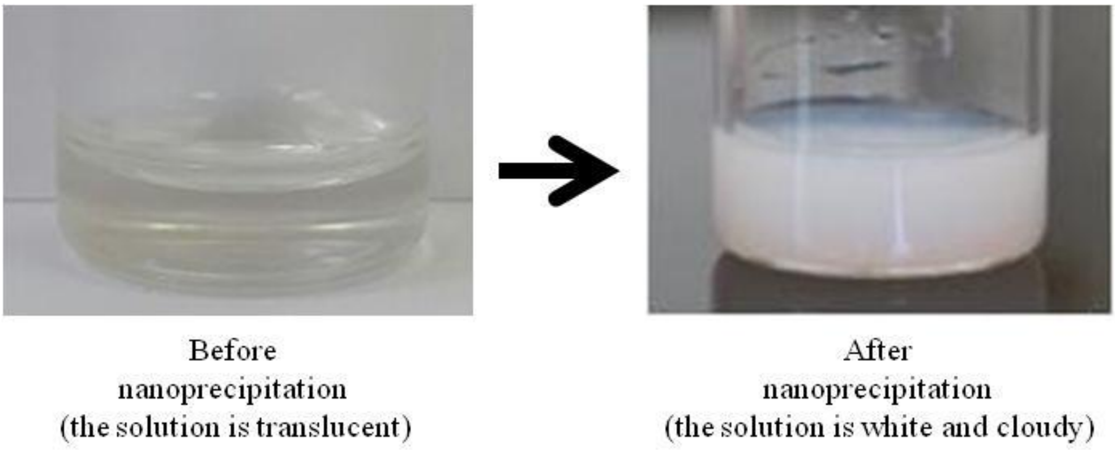

Supplementary Fig. S1: Nanoparticles formation. The bioconjugate solubilized in the solvent, is translucent while after the nanoprecipitation step in the water medium, the solution becomes white and cloudy suggesting auto-assembly into nanoparticles.

			Size (d.n...	% Intensity:	St Dev (d.n...
Z-Average (d.nm):	246,7	Peak 1:	262,1	100,0	66,50
PdI:	0,049	Peak 2:	0,000	0,0	0,000
Intercept:	0,944	Peak 3:	0,000	0,0	0,000
Result quality	Good				

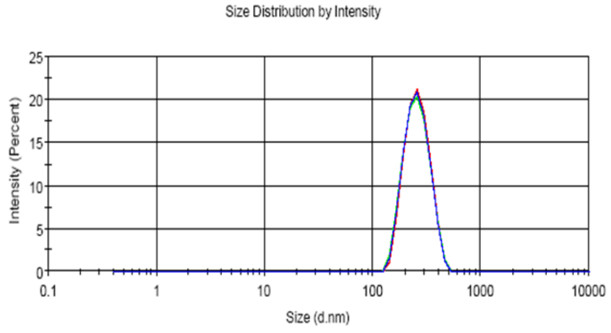

FaPenG NPs

			Size (d.n...	% Intensity:	St Dev (d.n...
Z-Average (d.nm):	192,6	Peak 1:	202,9	100,0	49,22
PdI:	0,031	Peak 2:	0,000	0,0	0,000
Intercept:	0,946	Peak 3:	0,000	0,0	0,000
Result quality	Good				

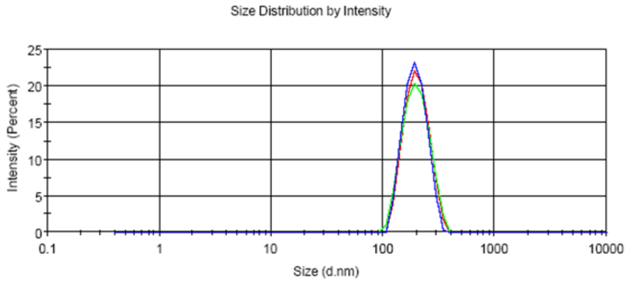

FaPenG-SB NPs

			Size (d.n...	% Intensity:	St Dev (d.n...
Z-Average (d.nm): 444,9		Peak 1:	478,9	100,0	142,5
PdI: 0,100		Peak 2:	0,000	0,0	0,000
Intercept: 0,954		Peak 3:	0,000	0,0	0,000
Result quality Good					

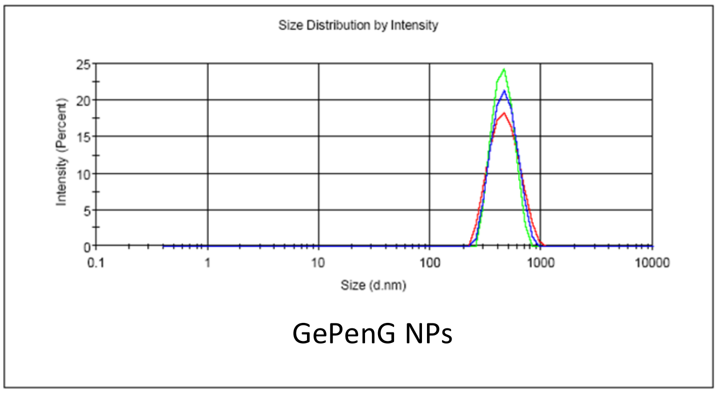

			Size (d.n...	% Intensity:	St Dev (d.n...
Z-Average (d.nm): 225,8		Peak 1:	238,6	100,0	60,07
PdI: 0,030		Peak 2:	0,000	0,0	0,000
Intercept: 0,955		Peak 3:	0,000	0,0	0,000
Result quality Good					

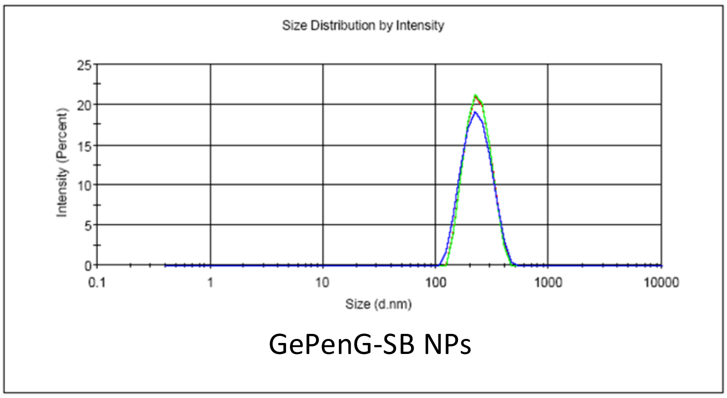

Supplementary Fig. S2: Nanoparticle size distribution measured by dynamic light scattering (DLS), using a Zetasizer Nano 6.12, (Malvern Instrument Ltd, Worcestershire, UK) for the FaPenG, FaPenG-SB, GePenG and GePenG-SB NPs. Each graph represents three successive measurements.

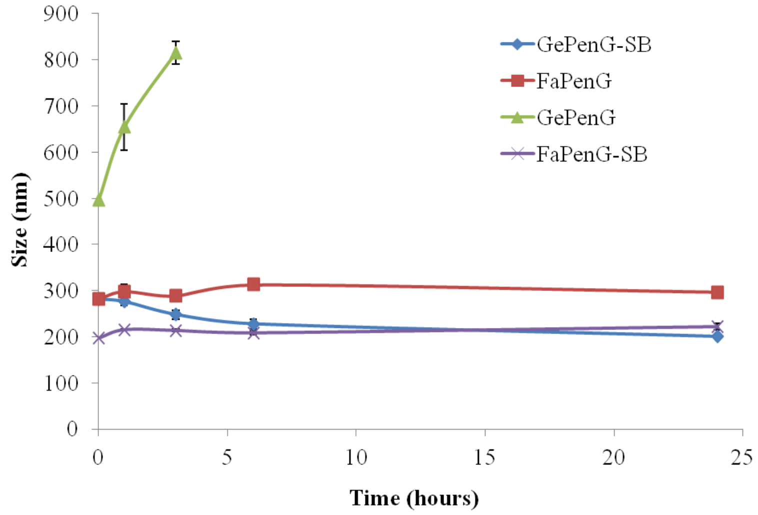

Supplementary Fig. S3: Stability of NPs during 24h in RPMI supplemented with 0.5% FBSd medium at 37°C. NPs with size over 850 nm were not reported on the graph.

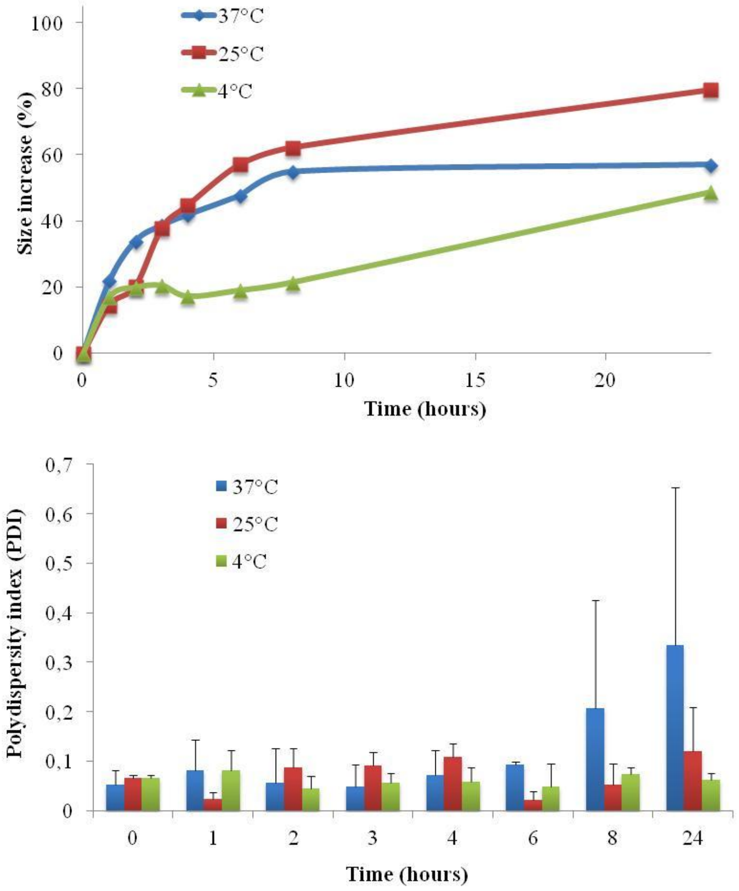

Supplementary Fig. S4: Stability of GePenG NPs during 24h in water at 37°C, 25°C and 4°C. Upper graph represent the percentage of increase in size and bottom graph represent the polydispersity index, both measured by DLS.

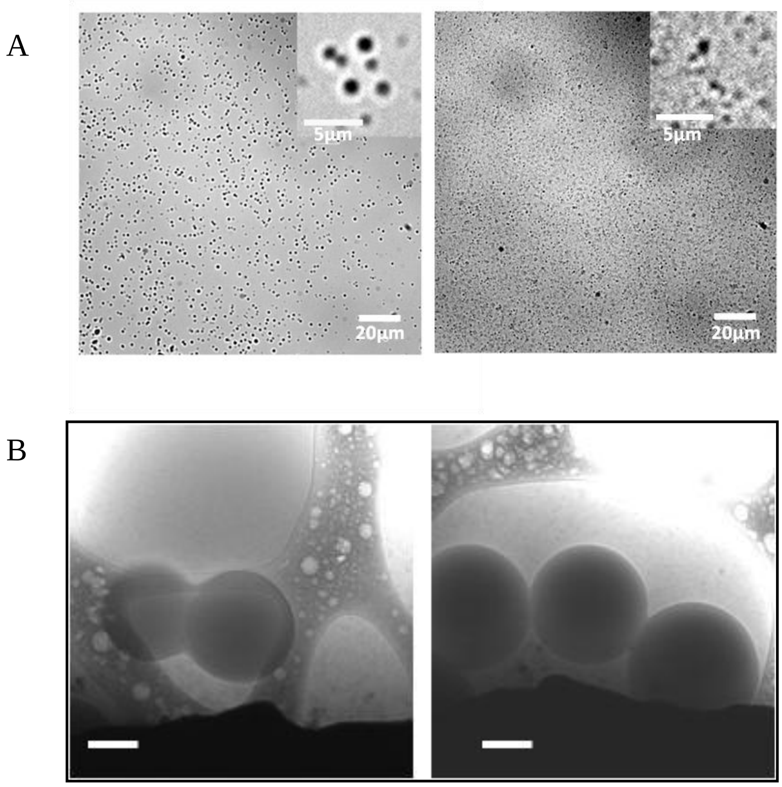

Supplementary Fig. S5: A) Optical microscopy of GePenG NPs (left side) and GePenG-SB NPs (right side) after 24h in water (37°C); scale bars = 20 µm. A magnification is placed on the right top corner; scale bars = 5 µm. B) Cryogenic transmission electron microscopy images of GePenG nanoparticles showing NPs coalescence. Scale bars = 100 nm.

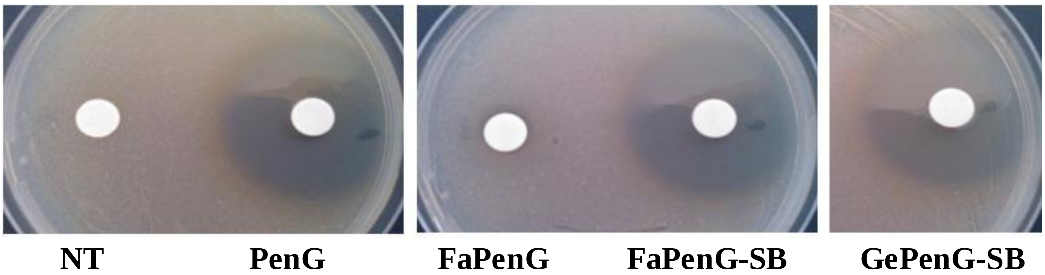

Supplementary Fig. S6. Disk diffusion antibiotic sensitivity assay. Each disk contains 10 μg.mL^{-1} equiv. of PenG, except for NT (no treated) which contains water.

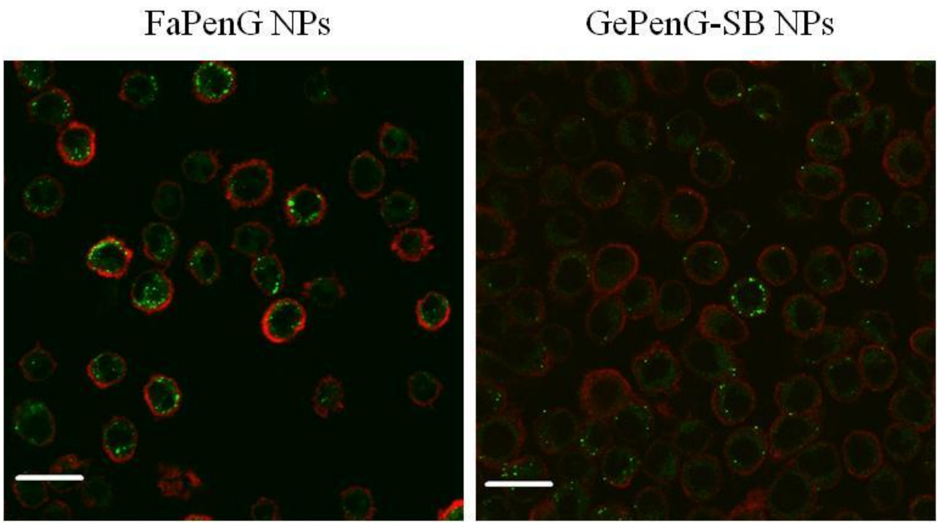

Supplementary Fig. S7: Cellular uptake of FaPenG::BC NPs and GePen-SB::BC NPs by RAW 264.7 cells, after 2 h incubation. Membranes are labeled in red with anti-CD11b antibody and NPs are green labelled. Scale bars= 20 µm.

A

B

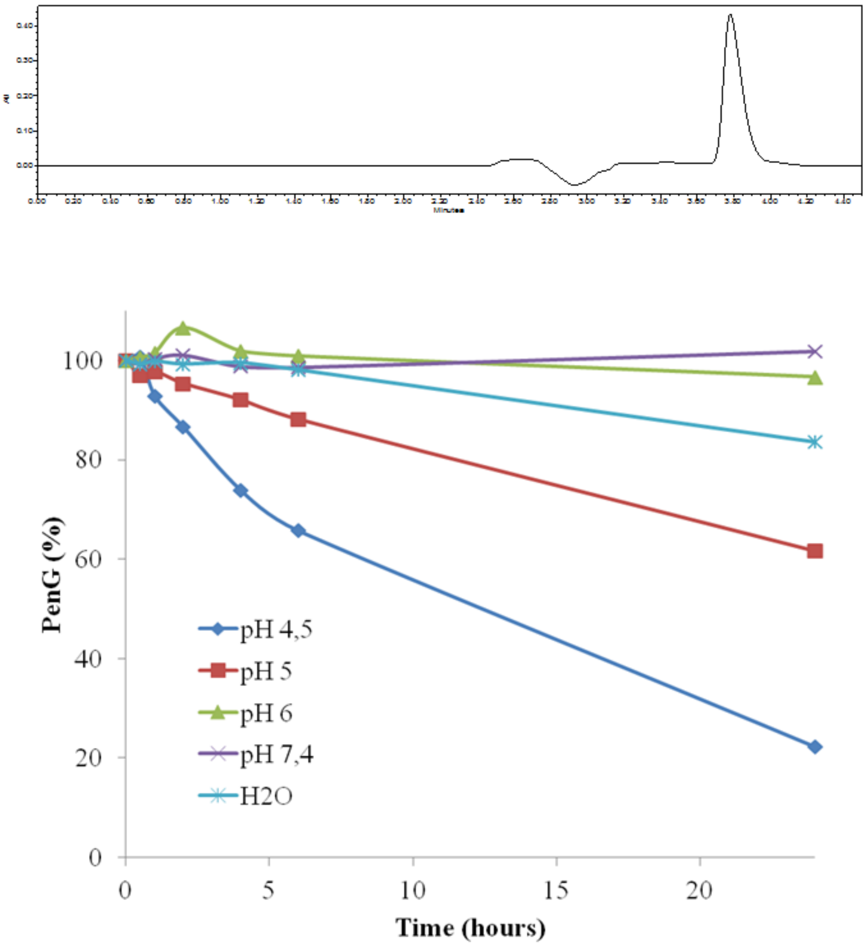

Supplementary Fig. S8: A) HPLC chromatogram of a PenG solution. Retention time = 3.8 min. B) Stability of PenG solution over time in different solutions: sodium citrate 0.1M, pH 4.5 ; sodium citrate 0.1M, pH 5 ; PBS at pH 6 ; PBS at pH 7.4 and in water.

Movie M1: GePenG-SB NPs on living RAW 264.7 cells after 2h treatment at 37°C.

2h hours after incubation with GePenG-SB NPs, the cells and nanoparticles are monitored using time-lapse imaging by fluorescence confocale. Images were obtained using different channels: phase contrast (to observe cell morphology and membrane integrity) and green fluorescence (to observe nanoparticles). Scale bar = 20 µm

Movie M2: FaPenG NPs on living RAW 264.7 cells after 2h treatment at 37°C.

2h hours after incubation with FaPenG NPs, the cells and nanoparticles are monitored using time-lapse imaging by fluorescence confocale. Images were obtained using different channels: phase contrast (to observe cell morphology and membrane integrity) and green fluorescence (to observe nanoparticles). Scale bar = 20 µm

Movie M3: GePenG-SB NPs on living RAW 264.7 cells after 5h treatment at 37°C.

5h hours after incubation with GePenG-SB NPs, the cells and nanoparticles are monitored using time-lapse imaging by fluorescence confocale. Images were obtained using different channels: phase contrast (to observe cell morphology and membrane integrity) and green fluorescence (to observe nanoparticles). Scale bar = 20 µm

Movie M4: FaPenG NPs on living RAW 264.7 cells after 5h treatment at 37°C.

5h hours after incubation with FaPenG NPs, the cells and nanoparticles are monitored using time-lapse imaging by fluorescence confocale. Images were obtained using different channels: phase contrast (to observe cell morphology and membrane integrity) and green fluorescence (to observe nanoparticles). Scale bar = 20 µm

Movie M5: RAW 264.7 cells treated with FaPenG-SB NPs over 24h at 37°C.

Time-lapse microscopy shows the cytotoxic effect of FaPenG-SB NPs on RAW 264.7 cells. Images were captured every 15 minutes from 3h to 24h after adding FaPenG-SB NPs. Images were obtained using different channels: phase contrast (to observe cell morphology) and green fluorescence (to observe nanoparticles).
